# First insights into the genetic diversity of *Mycobacterium tuberculosis *isolates from HIV-infected Mexican patients and mutations causing multidrug resistance

**DOI:** 10.1186/1471-2180-10-82

**Published:** 2010-03-17

**Authors:** Rocio Lopez-Alvarez, Claudia Badillo-Lopez, Jorge F Cerna-Cortes, Ivan Castillo-Ramirez, Sandra Rivera-Gutierrez, Addy C Helguera-Repetto, Diana Aguilar, Rogelio Hernandez-Pando, Sofia Samper, Jorge A Gonzalez-y-Merchand

**Affiliations:** 1 Hospital Regional "General Ignacio Zaragoza", Mexico City, Mexico; 2 Departamento de Microbiologia, ENCB-IPN, Mexico City, Mexico; 3 Instituto Nacional de Ciencias Medicas y Nutricion "Salvador Zubiran", Mexico City, Mexico; 4 Hospital Universitario Miguel Servet, Instituto Aragones de Ciencias de la Salud, Centro de Investigación Biomédica en Red Enfermedades Respiratorias, Zaragoza, España

## Abstract

**Background:**

The prevalence of infections with *Mycobacterium tuberculosis *(MTb) and nontuberculous mycobacteria (NTM) species in HIV-infected patients in Mexico is unknown. The aims of this study were to determine the frequency of MTb and NTM species in HIV-infected patients from Mexico City, to evaluate the genotypic diversity of the *Mycobacterium tuberculosis *complex strains, to determine their drug resistance profiles by colorimetric microplate Alamar Blue assay (MABA), and finally, to detect mutations present in *kat*G, *rpo*B and *inh*A genes, resulting in isoniazid (INH) and rifampin (RIF) resistance.

**Results:**

Of the 67 mycobacterial strains isolated, 48 were identified as MTb, 9 as *M. bovis*, 9 as *M. avium *and 1 as *M. intracellulare*. IS*6110*-RFLP of 48 MTb strains showed 27 profiles. Spoligotyping of the 48 MTb strains yielded 21 patterns, and 9 *M. bovis *strains produced 7 patterns. Eleven new spoligotypes patterns were found. A total of 40 patterns were produced from the 48 MTb strains when MIRU-VNTR was performed. Nineteen (39.6%) MTb strains were resistant to one or more drugs. One (2.1%) multidrug-resistant (MDR) strain was identified. A novel mutation was identified in a RIF-resistant strain, GAG → TCG (Glu → Ser) at codon 469 of *rpo*B gene.

**Conclusions:**

This is the first molecular analysis of mycobacteria isolated from HIV-infected patients in Mexico, which describe the prevalence of different mycobacterial species in this population. A high genetic diversity of MTb strains was identified. New spoligotypes and MIRU-VNTR patterns as well as a novel mutation associated to RIF-resistance were found. This information will facilitate the tracking of different mycobacterial species in HIV-infected individuals, and monitoring the spread of these microorganisms, leading to more appropriate measures for tuberculosis control.

## Background

Tuberculosis (TB) remains the most common opportunistic infection for people living with human immunodeficiency virus (HIV), and a leading cause of death in low and middle-income countries [1]. The number of new TB cases has tripled in countries where the incidence of HIV is high in the last two decades [2]. At least one-third of the 33.2 million people living with HIV worldwide are infected with TB and have up to 15% risk of developing TB every year, compared to those without HIV who have a 10% risk over their lifetime [3]. In Mexico, HIV-infected patients account for 1.0% of new TB cases [4]. In other developing countries, it has been reported that in HIV-infected patients, *Mycobacterium tuberculosis *(MTb) is not the only mycobacteria that causes disease, nontuberculous mycobacteria (NTM) have also been found in such patients [5,6]. In Mexico identification of mycobacterial species is generally based on clinical features, sometimes with the help of a positive acid-fast stain [7].

Since the discovery of polymorphic DNA in MTb, molecular typing of strains has become a valuable tool in TB epidemiological studies allowing investigators to track epidemics, detect new outbreaks, and achieve better knowledge of strain movement distinguishing between reinfection and relapse [8]. IS*6110 *restriction fragment length polymorphism (RFLP) typing of MTb has been used extensively in studies of TB transmission and is one of the most widely applied and standardized molecular typing methods [9,10]. Spacer oligonucleotide typing (spoligotyping) is another molecular genotyping technique; it is fast, robust, reliable, easy to perform, and cost-effective [11]. Spoligotyping is based on the analysis of the direct repeat (DR) loci, which are comprised of directly repeated sequences interspersed with non-repetitive spacer DNA [11]. This rapid PCR-based method allows the classification of strains into spoligotype families based on the presence or absence of spacer regions [12,13]. The most promising PCR-based methods are based on the analysis of multiple loci containing variable numbers of tandem repeats (VNTR) of different families of interspersed genetic elements, collectively called mycobacterial interspersed repetitive units (MIRU) [14,15]. Currently, the most commonly used version of this method (designated MIRU-VNTR) is based on the analysis of 12 loci [16]. Some authors have found that this method shows a discriminatory power equivalent to that of RFLP and for this reason it has been considered an alternative method to IS*6110*-RFLP for epidemiological studies [14,16,17]. One of the most alarming trends concerning TB is the emergence of drug-resistant MTb strains, which have become a worldwide health care problem [18]. The number of multidrug-resistant strains of MTb (MDR-TB), defined as resistant to at least isoniazid (INH) and rifampin (RIF), has been steadily increasing over the years, and several outbreaks have been reported [19,20]. The development of resistance to these two drugs reduces the efficacy of standard antituberculosis treatment to 77%. For this reason it is important to identify resistant strains as soon as possible to permit adjustments in treatment and minimize transmission of drug-resistant strains. Mutations in the catalase peroxidase gene (*kat*G) [21,22] and in a gene encoding the enoyl acyl carrier protein reductase (*inh*A) [23] have been found to account for 60 to 70% and 10 to 15% of INH-resistant MTb strains, respectively [24]. Mutations resulting in a single amino acid change within the 81-bp core region of the RNA polymerase β-subunit (*rpo*B) gene are found in 96% of RIF-resistant MTb strains [25].

The aims of this study were to determine the prevalence of mycobacterial species in HIV-infected patients from Mexico City and surrounding areas, to evaluate the genotypic diversity of the *Mycobacterium tuberculosis *complex (MTC) strains using IS*6110 *RFLP, spoligotyping and MIRU-VNTR, to determine their drug resistance profiles, and to detect mutations present in *kat*G, *inh*A and *rpo*B genes that lead to the selection of INH- and RIF-resistant strains.

## Results

### Mycobacteria prevalence in HIV-infected patients

In this study we characterized 67 mycobacterial strains isolated from HIV-infected patients, 85% of strains belonged to the MTC; 48 (71.6%) were MTb, 9 (13.4%) *M. bovis*, and the remaining 15% were NTM: 9 (13.4%) corresponded to *M. avium *and 1 (1.5%) to *M. intracellulare*. Thirty MTb strains (62.5%) were isolated from pulmonary specimens, while 8 of 9 *M. avium *strains (89%) were isolated from extrapulmonary specimens. Thirteen patients presented more than one site of infection (see Table 1).

**Table 1:** Genomic patterns of mycobacterial strains isolated from different clinical samples of the same patient.

Patient	Strain code	Clinical sample	Mycobacterial specie	Spoligotyping Share type (ST) number	RFLP cluster	MIRU-VNTR (code number)
1	IPN 24	Pleural effusion	MTb	ST42	16	23'3216153321
	IPN 25	urine	MTb	ST42	16	225225153323
						
2	IPN 29	Urine	MTb	ST732	12	220326153322
	IPN 31	Sputum	MTb	ST732	12	223326153322
						
3	IPN 64	Urine	MTb	ST119	9	223224153323
	IPN 67	Bronchoalveolar lavage fluid	MTb	ST119	10	23'3226133321
						
4	IPN 69	Urine	MTb	?^h^	23	332226153322
	IPN 70	Bronchoalveolar lavage fluid	MTb	?^h^	23	225223153322
						
5	IPN 74	Urine	MTb	ST50	1	223225123322
	IPN 75	Sputum	MTb	ST50	1	224325153324
						
6	IPN 10	Bone marrow	MTb	ST42	15	23'3226133321
	IPN 11	Sputum	MTb	ST42	15	225225153323
	IPN 12	Bronchoalveolar lavage fluid	MTb	ST42	15	225226143323
						
7	IPN 15	Pleural effusion	MTb	?^b^	●	223326133321
	IPN 16	Sputum	MTb	?^b^	●	223326133321
	IPN 17	Gastric fluid	MTb	?^b^	●	223226133322
						
8	IPN 85	Bone marrow	MTb	ST508	8	220225150020
	IPN 86	Bronchoalveolar lavage fluid	MTb	ST508	8	223225153321
	IPN 87	Sputum	MTb	ST508	8	220005153320
						
9	IPN 43	Pleural effusion	*M. bovis*	ST1306	ND	232224253322
	IPN 99	Lymph node	*M. bovis*	ST1625	ND	232224253322
						
10	IPN 56	Urine	*M. bovis*	ST409	ND	232224153323
	IPN 57	Sputum	*M. bovis*	ST409	ND	222224053320
	IPN 58	Cerebrospinal fluid	*M. bovis*	?^i^	ND	232224253322
						
11	IPN 46	Cerebrospinal fluid	*M. bovis*	ST683	ND	232224152320
	IPN 47	Cerebrospinal fluid	*M. bovis*	ST479	ND	332224153322
	IPN 48	Gastric fluid	*M. bovis*	?^j^	ND	232225153322
	IPN 49	Sputum	*M. bovis*	ST479	ND	332225153322
						
12	IPN 1	Bone marrow	*M. avium*	ND	ND	ND
	IPN 3	Gastric fluid	*M. avium*	ND	ND	ND
						
13	IPN 41	Bone marrow	*M. avium*	ND	ND	ND
	IPN 60	Lymph node	*M. avium*	ND	ND	ND

MTb, *M. tuberculosis*. ND, No determined, ?^b^, Spoligotype octal, 777743677760771, ?^h^, Spoligotype octal 777777660000131, ?^i^, Spoligotype octal 264063776776600, ?^j^, Spoligotype octal 677767777777600, ●, *M. tuberculosis *strains with zero-copy-numbers of IS*6110*.

### RFLP analysis

**Figure 1:**
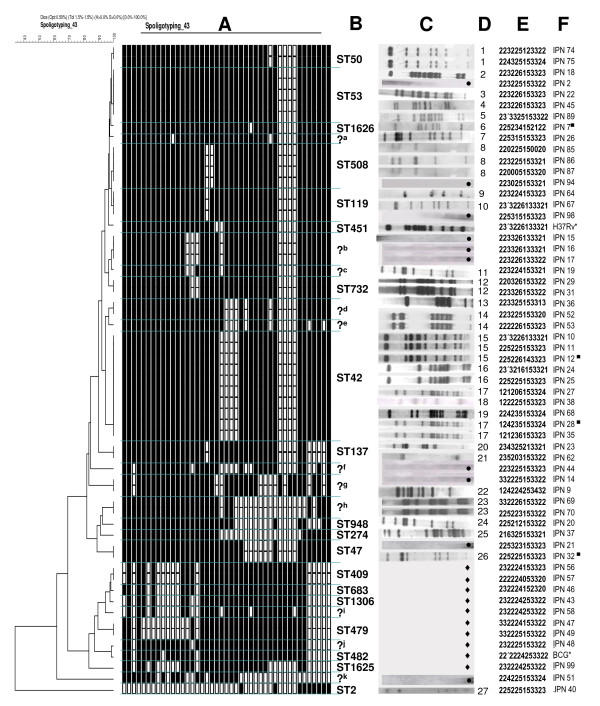
**Spoligotyping, IS*6110 *RFLP and MIRU-VNTR patterns of *M. tuberculosis *(MTb) and *M. bovis *strains isolated from HIV-infected patients**. The dendrogram is based on spoligotyping patterns. A) Spoligotyping patterns (binary representation); B) Shared-type (ST) number; C) IS*6110*-RFLP patterns; D) RFLP cluster; E)12 MIRU-VNTR patterns; F) Strain code. ?, new patterns of spoligotypes; ?^a^, Spoligotype octal 777577777720771, ?^b^, Spoligotype octal 777743677760771, ?^c^, Spoligotype octal 777747677760771, ?^d^, Spoligotype octal 777777705720771, ?^e^, Spoligotype octal 777777705720661, ?^f^, Spoligotype octal 677767604760621, ?^g^, Spoligotype octal 677777477413661, ?^h^, Spoligotype octal 777777660000131, ?^i^, Spoligotype octal 264063776776600, ?^j^, Spoligotype octal 677767777777600, ?^k^, Spoligotype octal 775346770000000; ●, *M. tuberculosis *strains with zero-copy-numbers of IS*6110*; H37Rv, *M. tuberculosis *H37Rv; BGC, *M. bovis *BCG; ♦, *M. bovis *strains. *, Reference strains used as controls. ■, INH-resistant MTb strains

RFLP analysis of all MTb strains was performed using IS*6110 *as probe. The IS*6110 *fingerprint patterns generated were highly variable. The number of IS*6110 *copies per strain varied from 0 to 14. Of IS*6110 *fingerprint patterns observed, 19 (39.6%) were unique, indicating epidemiological independence (Figure 1), and 10 strains (20.8%) lacked IS*6110 *(zero copy number). These patterns (zero IS*6110 *bands) were confirmed by performing a second RFLP analysis where MTb H37Rv was included as control strain. Additionally, 19 strains (39.6%) were clustered in 8 groups consisting of 2 to 3 strains with identical IS*6110 *RFLP patterns, presumably representing cases of recent transmission, and 16 (33.3%) strains presented IS*6110 *RFLP patterns with five bands or fewer.

### Spoligotyping

To determine lineage, the 57 strains (48 MTb and 9 *M. bovis*) from the MTC were spoligotyped and binary outcomes were compared with the shared type (ST) number and lineages and sublineages reported by Brudey et al [26]. Spoligotype analysis of 48 MTb strains yielded 21 patterns (Figure 1). Thirty-nine MTb strains (81.3%) were grouped into 12 clusters (2 to 10 strains per cluster) while 9 strains showed unique patterns. Thirty-four MTb strains showed 12 spoligotyping patterns that matched with: Shared-type (ST) number 2 (lineage name H2; n = 1), ST42 (LAM9; n = 10), ST47 (H1; n = 2), ST50 (H3; n = 2), ST53 (T1; n = 5), ST119 (X1; n = 3), ST137 (X2; n = 2), ST274 (U; n = 1), ST508 (T1; n = 4), ST732 (T1; n = 2), ST948 (H3; n = 1), and ST1626 (T1; n = 1). A further 14 MTb strains showed 9 patterns that did no exist in the SpolDB4.0 database (see question marks, Figure 1). Spoligotyping allows discrimination of MTb strains with low-copy-numbers of IS*6110 *(see Figure 1; for example, strains MEX-IPN 15, MEX-IPN 16, MEX-IPN17 and MEX-IPN 44). Nine *M. bovis *strains yielded 7 spoligotyping patterns; 5 unique patterns and 2 clusters with 2 strains in each one (Figure 1). The *M. bovis *spoligotyping patterns matched with ST409 (BOVIS2; n = 2), ST479 (BOVIS3; n = 2), ST683 (BOVIS2; n = 1), ST1306 (BOV; n = 1), ST1625 (BOVIS2; n = 1), and 2 new patterns were identified (Figure 1).

### MIRU-VNTR patterns

Clustering of MIRU-VNTR patterns by the UPGMA method showed a greater diversity of patterns in the mycobacterial strains studied. A total of 40 patterns were produced from 48 MTb strains, 5 clusters were identified (2 clusters with 4 and 3 strains, respectively, and 3 clusters with 2 strains in each). The remaining 35 strains showed unique patterns. Nine *M. bovis *strains produced a total of 7 patterns (Figure 1), 1 cluster was identified with 3 strains, while 6 strains presented unique patterns.

### Genomic diversity of MTb isolates

The discriminatory power of MIRU-VNTR typing was compared to that of IS*6110 *RFLP and spoligotyping by analyzing only MTb strains. Overall, MIRU-VNTR typing discriminated 40 different patterns (Figure 1); in comparison, only 27 different patterns were obtained with IS*6110 *RFLP and 21 patterns were obtained with spoligotyping. MIRU-VNTR typing performed even better than a combination of spoligotyping and IS*6110 *RFLP, which discriminated 36 patterns. The maximal discrimination was apparently achieved by combining MIRU-VNTR and IS*6110 *RFLP typing, resulting in 46 patterns.

Spoligotypes could often be distinguished by MIRU-VNTR typing; for instance, the single ST42 spoligotype corresponded to 9 distinct MIRU-VNTR genotypes (Figure 1). By contrast, just three of the MIRU-VNTR patterns (225225153323, 23'3226133321 and 225315153323) were distinguished by spoligotyping.

### Genomic patterns of mycobacterial strains isolated from the same patient

Identical spoligotyping and RFLP patterns were found among each set of strains in 7 out of 8 patients that were infected with more than one MTb strain (Table 1; patients 1, 2, 4-8). Only one patient (patient 3) had two strains that differed in both, RFLP and MIRU-VNTR typings, suggesting that, this particular patient was infected with two different strains of MTb. Regarding *M. bovis *strains, patients 9, 10 and 11 (Table 1) were infected with 2, 3 and 4 different strains according to their spoligotyping and MIRU-VNTR typing. Each of patients 12 and 13 were infected with two *M. avium *strains; but whether these are different strains remains to be determined.

### Phenotypic drug resistance testing

A total of 57 strains (48 MTb and 9 *M. bovis*) were subjected to colorimetric microplate Alamar Blue assay (MABA). Testing indicated that 9 *M. bovis *strains were susceptible to the 4 drugs tested, while 19 (39.6%) MTb strains showed resistance to one or more drugs (Table 2). Only one (2.1%) MTb strain was MDR, and 18 (95%) of them were resistant to STR. As none of *M. bovis *strains showed resistance to the 4 antibiotics tested, no further characterization was carried out on them. No phenotypic or genotypic drug resistance tests were carried out in NTM.

**Table 2:** Drug resistance of *M. tuberculosis *(MTb) strains isolated from HIV-infected patients

Drug resistance^a^	No. (%) of strains
***M. bovis*** Total strains	9 (100)
	
Non-resistant strains	9 (100)
	
***M. tuberculosis*** Total strains	48 (100)
	
Non-resistant strains	29 (60.4)
	
Strains resistant to one or more drugs	19 (39.6)
	
Resistance to one drug only	
STR	12 (25)
EMB	1 (2.1)
	
Resistance to more than one drug	
INH, STR	2 (4.2)
RIF, STR	1 (2.1)
STR, EMB	1 (2.1)
INH, STR, EMB	1 (2.1)
INH, RIF, STR, EMB	1 (2.1)

^a ^INH, isoniazid; RIF, rifampin; STR, streptomycin; EMB, ethambutol.

### Genotypic drug resistance testing

Mutations in *katG*, *inhA *and *rpoB *associated with resistance were found in 5 (10.4%) MTb strains. Our study shows that strains isolated from HIV-infected patients not only have mutations in regions of genes previously shown to be involved in drug resistance, but also have mutations that have not been previously reported. The nucleotide and amino acid changes identified in the drug resistant strains are shown in the Table 3. Among the INH-resistant strains, 3 strains had a mutation AGC → ACC at codon 315 of *kat*G gene (Ser → Thr), corresponding to the most common mutation found in INH-resistant strains [27,28]. The MDR strain had substitution mutations AGC → ACC (Ser → Thr) at codon 315 of *kat*G and TCG → TTG, at codon 531 of the *rpo*B gene, resulting in a predicted amino acid change of Ser → Leu. One RIF-resistant isolate had a mutation GAG → TCG (Glu → Ser) at codon 469 of the *rpo*B gene that has not been described previously. There was no correlation between the mutations and genotyping patterns of different strains in this study, i.e. the INH-resistant MTb strains (IPN7, IPN12, IPN28 and IPN32) had the same substitution mutation AGC → ACC (Ser → Thr) at codon 315 of the *kat*G gene, however they differ in the spoligotyping, IS*6110 *RFLP and MIRU-VNTR patterns (see Figure 1 and Table 3).

**Table 3:** Mutations found in *M. tuberculosis *(MTb) strains resistant to rifampin and isoniazid.

Rifampin			
Mutated *rpo*B codon	Specific mutation	Strain n	MIC (μg/ml)
531	TCG → TTG (Ser → Leu)^a^	1	>2
469	GAG → TCG (Glu → Ser)^b^	1	0.5
			
**Isoniazid**			
**Mutated** ***kat*G codon**	**Specific** **mutation**	**Strain** **n**	**MIC** **(μg/ml)**
315	AGC → ACC (Ser → Thr)^a^	3	>1
315	AGC → ACC (Ser → Thr)	1	1

^a ^Mutations found in the MDR *M. tuberculosis *strain

^b ^Mutation not described previously

## Discussion

In this study we analyzed 67 mycobacterial strains isolated from HIV-infected patients attending different hospitals in Mexico City. Diagnosis of mycobacterial infection in Mexico is based on clinical symptoms with Ziehl-Neelsen staining (AFB) being the only laboratory confirmation of infection currently in use. Many patients are treated for MTb purely on the basis of a positive AFB test and in most cases strains are not tested for NTM due to the procedure for this characterization being lengthy and expensive. The incomplete identification of mycobacterial species producing infection can have serious consequences, resulting in longer hospitalization times, increased risk of nosocomial infections and selection of MDR strains. Delayed diagnosis is a key factor contributing to the unnecessary deaths of many people living with HIV. More importantly proper identification of mycobacterial species causing infection leads to more appropriate antimicrobial treatment [29]. In agreement with results from a previous study by Molina-Gamboa et al [7], we found thatMTb was the most prevalent mycobacterial species identified in HIV-patient samples investigated in this study. Of the 9.27 million patients globally-infected with MTb in 2007, an estimated 1.37 million (14.8%) were HIV positive [30]. At least one-third of the 33.2 million people living with HIV worldwide are infected with TB and individuals infected with HIV are 20 to 30 times more likely to develop TB than those without the virus [2]. Although MTb is the most important etiological agent of TB, *M. bovis*, can also be considered a potential cause of human cases, especially in developing countries where control measures for bovine TB in cattle and/or milk dairy products are not always satisfactory [31]. With the advent of HIV, bovine TB represents an additional risk for HIV-infected patients. Importantly, pulmonary or extrapulmonary TB caused by *M. bovis*, may be underestimated due to the fact that the resulting infection is clinically indistinguishable from that caused by MTb. In this study 13.4% of strains isolated were identified as *M. bovis*. Our results are consistent with those reported by Cicero et al [32], who also identified *M. bovis *in extrapulmonary samples (13.75%) from HIV-infected patients in Mexico. In an earlier study, Molina-Gamboa et al [7] identified *M. bovis *in 4.6% of patients with HIV using only biochemical tests.

Although in the past two decades NTM infections have been regarded as a growing concern, mainly as a result of the AIDS epidemic, these microorganisms were first recognized in the 1950s when the prevalence of TB fell after the introduction of antimycobacterial therapy [33]. NTM produce both pulmonary and extrapulmonary disease in both immunocompetent and immunocompromised subjects [33]. In this study, 15% of isolated mycobacterial strains were NTM. The mycobacteria identified in this study belonged to the MAC complex: *M. avium*-*M. intracellulare*, findings which are consistent with those reported by Molina-Gamboa et al [7], who identified these mycobacteria as the second most prevalent acid-fast bacilli isolated from HIV-infected patients in Mexico. Countries with limited resources like Mexico do not identify mycobacteria by culture and molecular techniques and because of this infections caused by NTM are under diagnosed or misdiagnosed. This study emphasizes the need for molecular identification of NTM in HIV-infected patients.

RFLP analysis based on IS*6110 *insertion is used to define clusters of MTb strains with identical DNA fingerprints. However, to the best of our knowledge, there have been no studies in Mexico that have used IS*6110 *RFLP analysis to characterize MTb strains isolated from HIV-infected patients. Using this method we showed wide genetic variability in Mexican strains (27 patterns from 48 MTb strains). Our results are similar to those reported in countries like Tanzania where Yang et al [34] obtained 60 patterns from 68 MTb clinical strains and In Switzerland, where Strässle et al [35] identified 40 different patterns from 52 MTb strains isolated from HIV-infected patients. Our findings differ from reports of the numbers of different MTb strains isolated from non-HIV population within endemic regions, where it has been shown that variability in IS*6110 *patterns is low [36]. The contrasting wide diversity of MTb strains from HIV-infected patients found in Tanzania, Switzerland and now in Mexico, might be explained by these patients having a deficient immune system, and thus providing the perfect habitat for the development of infection regardless of mycobacterial virulence [34].

In the present study we identified 16 MTb strains (33.3%) with five or fewer copies of IS*6110*; 10 of these (20.8%) lacked IS*6110*. MTb strains with low IS*6110 *copy number have been more frequently isolated from Asian patients than from European patients. For example, 56% of the strains collected from India and 29 to 37.5% of the strains collected from Vietnam, Thailand and Malaysia contained five or fewer IS*6110 *elements [37,38], whereas the frequencies of low-copy-number strains in Denmark and France were 11% and 8%, respectively [39,40]. In the United States, analysis of strains from Texas, California, and Colorado reported 25% containing fewer than six IS*6110 *copies [41]. The reports of the incidence of strains with low copy number insertions from the United States are closer to the incidence of the Mexican strains isolated in our work.

In this study, 48 MTb strains produced 21 spoligotyping patterns, while 9 *M. bovis *produced just 7 patterns. Quitugua et al [42] had reported the spoligotype 777776777760601 (ST137) in 63 patients from Texas, this pattern was identified in 2 strains in our study. Likewise, the octal 777776777760771 (ST119) which was identified in 89 patients who live on the border of Mexico (Tamaulipas) and United States (Texas), was identified in 3 strains in this study. Other octals found by Quitugua et al and also in our work, were 777777777760771 (ST53) and 777777607760771 (ST42), confirming that there are some strains of MTb circulating between Mexico and United States. The spoligotypes ST42, ST47, ST50 and ST53 identified in this study, have been found in others countries including Brazil, South Africa and Poland [43-45], suggesting that these strains might be circulating worldwide. Furthermore, the ST53 spoligotype has also been isolated from Egyptian mummies [46]; this spoligotype is one of the most common patterns and, according to a hypothesis about the evolution of MTb strains by loss of DRs [47], close to the origin of development of mycobacterial diversity.

The ST683 spoligotype found in *M. bovis *strains isolated in this study has also been found in cattle from Juarez City and Chihuahua (Mexico) [48] and has been frequently isolated from cattle in Australia, Argentina, England, France and Ireland [49-53]. The pattern of transmission of *M. bovis *to HIV-infected patients is still under study; however, the identification of the same spoligotype patterns in both cattle and HIV-infected patients indicates that, as is generally accepted, ingestion of contaminated milk or dairy products is the most probable origin of infection [31].

This study is the first in Mexico where genetic diversity of mycobacterial strains has been evaluated using MIRU-VNTR. The 48 MTb strains investigated in this report produced 40 distinct patterns by MIRU-VNTR while 9 *M. bovis *strains produced 7. Analysis of these results showed that most of these patterns were unique, consistent with other studies conducted in Singapore and Belgium, where there was wide variability in MTb strains [54,55]. As expected, most of clusters based on spoligotyping or low IS*6110 *copy number fingerprinting could be distinguished by MIRU-VNTR. Additionally, in strains isolated from HIV-infected patients, 4 MIRU (4, 20, 23 and 31) were showed to have a different pattern compared with those occurring in the population without HIV; MIRU 4 and 31 in strains isolated from HIV-infected patients presented with low polymorphism, while those identified from individuals without HIV have a high polymorphism. By contrast, MIRU 20 and 23 of strains from HIV-infected patients have a high polymorphism, while those from individuals without HIV showed a low polymorphism [16,56,57]. These differences might be useful for the differentiation and classification of strains that can only infect HIV patients.

Some authors have found that MIRU-VNTR based on a 12-loci set (MIRU-12) format have limitations in its discriminatory power [58-60]. Recently, two MIRU-VNTR formats (MIRU-15 and MIRU-24) have been developed to improve the discriminatory power of MIRU-12 [61], and found a better discriminatory power using the set of 15-loci (MIRU-15) with 825 MTb isolates. However, in our study, the MIRU-12 allowed us to demonstrate a high genetic diversity in mycobacterial strains belonging to the MTC; in order to get a more definitive answer to this matter, more genotyping analysis should be carried out with MTb strains from different origins.

Since all isolates were collected from HIV-infected patients, we suggest to analyze MTC strains from non VIH-infected patients from the same region in order to enhance the significance of our results.

MDR TB is an increasing problem worldwide [62]. Infection with MDR MTb is associated with significant mortality [18], and has resulted in a number of serious outbreaks [63]. Colorimetric microplate Alamar Blue assay (MABA) assays demonstrated that all isolated *M. bovis *strains were susceptible to the antibiotics tested. On the other hand, 19 (39.6%) isolated MTb strains were resistant to one or more antibiotics. These results are very close to those obtained by Peter et al [64], who demonstrated that 41% of the MTb strains isolated from patients from Baja California (Mexico) were resistant to at least one antibiotic. Our study showed that 2.1% of the strains we identified were MDR, confirming the incidence of MDR TB in Mexico already reported by the WHO [4]. The highest proportions of strains were resistant to STR, as has also been reported to be the case in Africa for both HIV-infected and patients without HIV [65,66]. Due to the importance of INH and RIF, which are the most effective antibiotics against TB, we determined the mutations that lead to the selection of resistant strains in our study. Three INH-resistant strains showed a mutation AGC → ACC (Ser → Thr) at codon 315 of *kat*G gene, a finding consistent with several studies, which have shown that this mutation is the most frequently associated with this resistance [27,67]. In our country, this mutation seems to be as frequent [27,28], as in other countries such as Russia and Brazil [20,67]. In this study, no correlation was found between genotypic drug resistance and genotypic patterns, findings which were consistent with those previously reported for MTb strains isolated in both HIV-infected and non HIV-infected patients [27,66,67]. On the other hand, one RIF-resistant isolate had a mutation GAG → TCG (Glu → Ser) at codon 469 of *rpo*B gene, which has not been previously described, and which should be the object for further study of other strains in Mexico.

## Conclusions

This is the first molecular analysis of mycobacteria isolated from HIV-infected patients in Mexico, which describe the prevalence of different mycobacterial species in this population. Using a combination of different molecular techniques a high genetic diversity of MTb strains was identified. New spoligotypes and MIRU-VNTR patterns as well as a novel mutation associated to RIF-resistance were found. This information will facilitate the tracking of different mycobacterial species in HIV-infected individuals, and monitoring the spread of these microorganisms, leading to more appropriate measures for TB control in these patients.

## Methods

The present experimental research that is reported in the manuscript has been performed with the approval of the Ethical Committee of the Escuela Nacional de Ciencias Biologicas, IPN, Mexico and carried out within an ethical framework.

### Mycobacterial strains

Sixty seven Mycobacterial strains were isolated from 55 HIV-infected patients at different National Health Service hospitals in Mexico City (General Hospital of Mexico, Hospital Regional "General Ignacio Zaragoza", National Medical Center "Siglo XXI" and National Medical Center "La Raza") between January and December 2006. All patients were on treatment with antiretroviral medication and their CD4 lymphocyte counts varied from 100 to 300 cells/mm^3^. According the WHO data [68], the 55 HIV/TB patients corresponded aprox. to 21% of the total patients attended in México in 2006.

Mycobateria were isolated from sputum, bronchoalveolar lavage fluid, cerebrospinal fluid, urine, bone marrow, lymph node, pleural effusion, ascitic fluid, tissue biopsy, pericardial fluid, gastric fluid. Isolation and identification of mycobacteria was carried out by the Microbiology service of each hospital using acid-fast staining (AFB).

Thirty-one (46.3%) strains were isolated from sputum and 36 (53.7%) from extrapulmonary clinical samples.

### Identification of mycobacterial species

Mycobacterial genomic DNA was isolated by guanidinium chloride extraction [69]. The identity of the 67 isolated strains was confirmed by PCR as described previously [70]. Briefly, a multiplex PCR reaction was performed to identify the genus of *Mycobacterium *and *M. bovis *species, and a second PCR reaction was carried out to determine if a clinical isolate belonged to the *M. tuberculosis *complex. Nontuberculous mycobacteria (NTM) were identified by sequencing the V2 region of the 16S rRNA gene [71], using the RAC8 primer (5'-CACTGGTGCCTCCCGTAGG-3'), and ABI PRISM 310 genetic analyzer (Perkin-Elmer). All sequences were analyzed by BLAST [72].

#### DNA fingerprinting

Mycobacterial strains belonging to MTC were subjected to spoligotyping, MIRU-VNTR analysis, phenotypic and genotypic drug resistance tests. Only MTb strains were subsequently subjected to restriction fragment length polymorphism (RFLP) analysis.

### IS*6110 *RFLP

IS*6110 *fingerprinting was performed as described previously [73]. Briefly, genomic DNA from each MTb isolate (2 μg) was digested with PvuII. Fragments were separated by electrophoresis on agarose gels, denatured and transferred by Southern blotting to nylon membrane. Hybridization was performed with a chemiluminescence-labeled 521-bp IS*6110 *fragment. MTb H37Rv was used as control.

### Spoligotyping

This technique was carried out as described previously [11]. The DR region was amplified using oligonucleotides DRa (5'-GGTTTTGGGTCTGACGAC-3', biotinylated) and DRb (5'-CCGAGAGGGGACGGAAAC'-3'). Labeled amplification products were used as a probe for hybridization with 43 synthetic spacer oligonucleotides covalently bound to a membrane (Isogen Biosciences B.M., Maarssen, The Netherlands). Each oligonucleotide corresponded to a known spacer sequence. PCR product bound after hybridization was detected by streptavidin-horseradish peroxidase-enhanced chemiluminescence (Amersham, Little Chalfont, England) according to manufacturer's instructions. Spoligotypes were reported using an octal code [74]. Analysis of spoligotypes was performed using Bionumerics software version 5.5 (Applied Maths, Kortrijk, Belgium). MTb H37Rv and *M. bovis *BCG were used as controls.

### MIRU-VNTR analysis

MIRU-VNTR typing was performed as described previously [16]. Bacteria were resuspended in 200 μl milli-Q water, boiled for 10 min, and cooled on ice or 5 min. Supernatant from bacterial lysates (2 μl) was added to MIRU-PCR mix (0.1 μl of HotStart *Taq *DNA polymerase (0.5 U) (Qiagen) with 4 μl of Q-solution, 0.5 mM each dATP, dCTP, dGTP, dTTP, 2 μl of PCR buffer, variable concentrations of each primer, and 1.5 mM MgCl_2_) in 20 μl final volume. The oligonucleotides used corresponded to the flanking regions of the 12 polymorphic MIRU-VNTR loci identified in the *M. tuberculosis *H37Rv genome as described by Supply et al [75]. PCR reactions were performed in a PXE0.2 thermo cycler (Thermo Electron Corporation) following a protocol of: 95°C for 15 min, followed by 40 cycles of 94°C for 1 min, 59°C for 1 min, and 72°C for 1.5 min, with a final extension at 72°C for 10 min. PCR fragments were analyzed on a 2100 Bioanalyser (Agilent Technologies). Genotypes were expressed as numerical code representing the number of MIRU-VNTR in each loci. A dendrogram was constructed by the unweighted-pair group method using average linkages (UPGMA) after pairwise comparison of strains by calculation of the Jaccard index.

### Phenotypic drug resistance testing (PDRT)

Strains were tested for PDR by colorimetric microplate Alamar Blue assay (MABA) in 96-well flat-bottom plates (Nunc International, Rochester, NY, USA) as described by Franzblau et al [76], with some modifications [77]. Briefly, cultures in exponential growth phase were diluted with sterile Middlebrook 7H9 broth supplemented with 10% OADC (oleate-albumin-dextrose-catalase) until they reached McFarland tube no. 1 turbidity, then further diluted 1:10. Streptomycin (STR), isoniazid (INH), ethambutol (EMB) and rifampin (RIF) were obtained from Sigma Chemical (USA). One hundred microliters of MTb inoculum was incubated in medium without drug or with drugs in the following concentration ranges: INH, 1 to 0.031 μg/ml; RIF, 2 to 0.062 μg/ml; STR, 8 to 0.25 μg/ml; and EMB, 32 to 1 μg/ml. Following incubation for 5 days at 37°C indicator solution (20 μl of Alamar Blue [Trek, OH, USA] and 12 μl of sterile 10% Tween 80) was added to control inoculi without drugs and plates were incubated at 37°C for a further 24 h. If the medium in control inoculi turned pink, subsequently indicator solution was added to inoculi that had been incubated with drugs and after 24 h incubation the colour of all the samples was recorded. Wells remaining blue were scored as "negative growth". The minimal inhibitory concentration (MIC) was defined as the lowest drug concentration that prevented colour change. If by day 6 no change was recorded in the drug-free control, the plate was incubated for a further 3 days; if control inoculi were still negative, a second control inoculum was used (day 9) and the whole procedure was repeated. MTb H37Rv was included as control strain. An isolate was considered drug resistant when the MIC was higher than 0.25 μg/ml for INH, 0.25 μg/ml for RIF, 2.0 μg/ml for STR, and 8 μg/ml for EMB [77]. Multidrug resistance (MDR) was defined in accordance with standard criteria of resistance to both INH and RIF at least.

### Genotypic drug resistance testing

Multiplex PCR [78] was used to detect the AGC → ACC (serine to threonine) mutation in codon 315 of the *kat*G gene (primers: katg0F 5'-GCAGATGGGGCTGATCTACG-3' and R315 mut 5'-TCCATACGACCTCGATGCCAG-3') and to detect -15 C-to-T and -14 G-to-A substitutions (primers: mabAF 5'-CGAAGTGTGCTGAGTCACACCG-3' and inhARmut 5'-AGTCACCCCGACAACCTATTA-3') within the promoter region of the *mab*A-*inh*A operon. Following PCR, DNA from resistant strains with these mutations yielded 296-bp and/or 146-bp PCR products. Bacterial DNA (50-100 ng) was used as a template in PCR reactions with pure*Taq *Ready-To-Go PCR bead kit (Amersham Biosciences, Piscataway, N.J.). The PCR mix consisted of 10 mM Tris-HCl (pH 9), 50 mM KCl, 1.5 mM MgCl_2_, a 200 μM of each deoxynucleotide, 2.5 U of pure*Taq *DNA polymerase and PCR primers (200 mM for *kat*G and 400 mM for *mab*A-*inh*A) in a final volume of 25 μl. Reactions were performed in a PXE0.2 thermo cycler (Thermo Electron Corporation) starting with a 5 min denaturation at 95°C, followed by 30 cycles of 95°C for 1 min, 68°C for 1 min and 72°C for 45 s, with a final extension at 72°C for 10 min. PCR products were resolved by electrophoresis in 2% agarose gels and detected by staining with ethidium bromide.

Rifampin resistant isolates were detected by amplification of a 437 bp fragment incorporating the *rpo*B-hotspot region from bacterial DNA using primers rpoB-F1 and rpoB-R1 as described previously [25]. PCR products were sequenced using a 310 ABI PRISM sequencer (Applied Biosystems). Sequences were compared to the sequence of the rpoB-hotspot from wildtype bacteria using BLAST [72].

## Authors' contributions

Conceived and designed the experiments: JFC-C, JAG-y-M. Performed the experiments: RL-A, CB-L, IC-R, SR-G, ACH-R, DA. Analyzed the data: JFC-C, RH-P, SS, JAG-y-M. Write the paper: JFC-C, SS, JAG-y-M. All Authors have read and approved the final manuscript.
